# Soluble Isoforms of PD-1 and PD-L1 in Non-Small Cell Lung Cancer: Correlation with Tumor Stage, Longitudinal Analysis and Prognostic Implications

**DOI:** 10.3390/jpm16040203

**Published:** 2026-04-04

**Authors:** Konstantinos Vachlas, Dimitra Grapsa, Stylianos Gaitanakis, Anna Papadopoulou, Paraskevi Moutsatsou, Nikolaos Syrigos, Ioannis P. Trontzas

**Affiliations:** 1 Thoracic Surgery Department, Sotiria Hospital of Respiratory Diseases, 11527 Athens, Greece; 2 3rd Department of Internal Medicine, National and Kapodistrian University of Athens, Sotiria Hospital of Respiratory Diseases, 11527 Athens, Greece; dimgrap@yahoo.gr (D.G.);; 3 Thoracic Surgery Department, University Hospitals Bristol and Weston Foundation Trust, Bristol Royal Infirmary, Bristol BS2 8HW, UK; stylianos.gaitanakis@uhbw.nhs.uk; 4 Department of Clinical Biochemistry, National and Kapodistrian University of Athens, University General Hospital Attikon, 12462 Athens, Greece

**Keywords:** non-small cell lung cancer, soluble PD-L1, soluble PD-1, biomarkers, immunotherapy, kinetics, prognosis

## Abstract

**Background:** Soluble immune checkpoint molecules, including soluble PD-1 (sPD-1) and soluble PD-L1 (sPD-L1), have emerged as potential minimally invasive biomarkers in non-small cell lung cancer (NSCLC). However, their diagnostic, kinetic, and prognostic significance across different disease settings remains unclear. This prospective study evaluated baseline levels, longitudinal fluctuations, and clinical associations of sPD-1 and sPD-L1 in early- and advanced-stage NSCLC. **Methods:** Three cohorts were prospectively enrolled: early-stage NSCLC patients undergoing curative surgery (n = 25), advanced-stage NSCLC patients receiving pembrolizumab-based immunotherapy (n = 55), and non-oncological controls (n = 16). Serum sPD-1 and sPD-L1 were measured by ELISA at baseline and at four months post-surgery (early stage) or six months post-treatment (advanced stage). Baseline comparisons, longitudinal changes, correlation with tumor PD-L1 expression (TPS), and associations with recurrence (early stage) or 6-month objective response (advanced stage) were assessed. **Results:** Baseline sPD-1 and sPD-L1 levels did not differ significantly among controls, early-stage, and advanced-stage cohorts. In early-stage patients, sPD-L1 increased post-operatively (*p* = 0.006) while sPD-1 decreased (*p* < 0.001). In advanced-stage disease, sPD-1 declined during immunotherapy (*p* < 0.001), whereas sPD-L1 remained unchanged (*p* = 0.37). Baseline levels and continuous percent changes were not predictive of most outcomes. However, a ≥20% postoperative increase in sPD-L1 was strongly associated with recurrence in early-stage NSCLC (OR = 10.29; 95% CI: 1.40–215.20; *p* = 0.019). No sPD-1/PD-L1 metric predicted response in advanced disease. Baseline sPD-L1 showed no correlation with tumor PD-L1 expression (ρ = −0.09, *p* = 0.53) in the advanced-stage cohort. **Conclusions:** sPD-1 and sPD-L1 demonstrate distinct kinetic patterns across NSCLC settings. A postoperative >20% surge in sPD-L1 may identify early-stage patients at elevated risk of recurrence, whereas soluble checkpoints were not predictive of treatment response in advanced disease. These findings support further investigation of soluble checkpoint dynamics as complementary biomarkers in NSCLC management in larger cohorts.

## 1. Introduction

Immunotherapy with immune checkpoint inhibitors (ICIs) has transformed the therapeutic landscape of non-small cell lung cancer (NSCLC). Initially approved for the treatment of advanced and metastatic disease, ICIs have since expanded into the early-stage setting, where peri-operative approaches have become standard of care for selected patients [1,2].

Despite their unprecedented benefits, most patients ultimately experience primary or acquired resistance. In non-oncogene-addicted metastatic NSCLC, median overall survival (OS) ranges from 17 to 26 months in randomized trials evaluating ICIs alone or in combination with chemotherapy [3,4]. Treatment benefit is strongly dependent on PD-L1 expression, with patients whose tumors exhibit high PD-L1 levels (tumor proportion score, TPS ≥ 50%) deriving the greatest benefit—achieving 5-year survival rates of up to 32%, with 82% of those completing 2 years of therapy remaining alive at 5 years [5]. However, while high PD-L1 expression enriches for ICI responders, lower PD-L1 levels (<50%) lack sufficient precision to reliably distinguish patients who will benefit from therapy from those who may be exposed to unnecessary toxicity.

Conversely, in early-stage clinical trials, ICIs have demonstrated promising efficacy regardless of baseline PD-L1 expression [2,6,7]. In this setting, complete pathological response (pCR) is associated with favorable long-term outcomes; however, there are currently no validated biomarkers for prognostic stratification or prediction of immunotherapy response among patients who do not achieve pCR.

The limitations of PD-L1 expression and other tissue-based biomarkers—such as spatial heterogeneity, temporal variability, and the constraints of small biopsy samples—underscore the need for more accurate and dynamic predictors across disease stages. Peripheral blood-based monitoring strategies have emerged as appealing adjunctive tools, offering a minimally invasive, repeatable approach for assessing treatment efficacy and detecting imminent progression or recurrence [8]. Recent investigations have turned to soluble isoforms of immune checkpoint proteins, including soluble PD-1 (sPD-1) and soluble PD-L1 (sPD-L1), as potential biomarkers. Their circulating levels may reflect prognostic information in NSCLC and may aid in predicting ICI response or risk of disease recurrence, as suggested by several preliminary studies [9,10,11]. Soluble forms of PD-1 and PD-L1 arise through multiple biological processes, including alternative mRNA splicing and proteolytic cleavage of their membrane-bound counterparts, as well as release from tumor and immune cells [8,12,13]. Rather than representing passive bioproducts, these circulating isoforms may retain functional activity with the PD-1/PD-L1 signaling axis. Experimental data suggest that sPD-L1 can bind to PD-1 on T-cells and contribute to systemic suppression, whereas sPD-1 may interfere with PD-1/ligand interactions and thereby modulate checkpoint signaling, potentially enhancing T-cell activity [8,12,13]. This emerging evidence supports the role of soluble checkpoint molecules in the modulation of systemic immune dynamics beyond the tumor microenvironment.

Herein, we sought to evaluate the clinical utility of two soluble checkpoint proteins, sPD-L1 and sPD-1, across different NSCLC settings. Specifically, we prospectively assessed their kinetics before and after treatment, examined their relationship with disease presence and stage, explored their correlation with tumor PD-L1 expression, and investigated their prognostic implications.

## 2. Methods

### 2.1. Study Population

All consecutive patients with histologically-confirmed early-stage NSCLC who presented to the Thoracic Surgery Department of Sotiria Hospital of Respiratory Diseases (Athens, Greece) and were candidates for upfront surgical resection, as well as patients with advanced-stage NSCLC who presented at the Oncology Unit of the 3rd Department of Internal Medicine, National and Kapodistrian University of Athens (same institution) with unresectable disease, were prospectively screened for enrollment.

Advanced-stage patients were enrolled upon confirmation of eligibility for first-line treatment with ICIs. Patients who had received any prior systemic anticancer therapy before enrollment were excluded from both cohorts. For the advanced-stage cohort, patients harboring actionable mutations eligible for first-line targeted treatment (e.g., *EGFR* mutation, *ALK* rearrangement) were also excluded. Prior or concurrent radiotherapy, as well as concurrent chemotherapy, was permitted according to the national guidelines.

Patients without NSCLC who were admitted to the Thoracic Surgery Department for non-oncological reasons were non-consecutively enrolled and comprised the control cohort.

All participants provided written informed consent. The study was conducted in accordance with the Declaration of Helsinki and was approved by the Institutional Scientific Board (protocol number: 26885/15-11-19). De-identified raw data are provided in Supplements File S1.

A subset of patients (advanced-stage cohort) has been included in a previously published analysis focused on survival outcomes; the current study addresses distinct kinetic and response-based endpoints with different sampling timepoints. Comparative evaluation with the prior publication is provided in the Section 4.

### 2.2. Demographics and Clinical Follow-Up

Baseline characteristics were recorded for each participant at enrollment. Age and sex were documented for all three cohorts. For NSCLC patients, the histological subtype and clinical stage were reported.

For the early-stage cohort, the type of surgery, pathological stage, and administration of adjuvant chemotherapy were also recorded. For the advanced-stage cohort, Eastern Cooperative Oncology Group Performance Status (ECOG PS), PD-L1 expression (tumor proportion score, TPS), line of treatment, and administered agents were documented.

Clinical follow-up for the early-stage patients was performed for up to five years or until disease recurrence, whichever occurred first. Advanced-stage patients were followed for six months, and the best objective response was recorded. Treatment response was evaluated according to Response Evaluation Criteria in Solid Tumors (RECIST) version 1.1 [14], and categorized as complete response (CR), partial response (PR), stable disease (SD), or progressive disease (PD).

### 2.3. Sample Collection and Measurement of sPD-1 and sPD-L1

Peripheral venous blood samples for soluble checkpoint measurements (sPD-1 and sPD-L1) were collected at baseline (prior to surgery or before the first cycle of immunotherapy, respectively) and again at four months post-surgery for the early stage (to also capture the potential effect of adjuvant chemotherapy) and at six months post-treatment for the advanced-stage cohort.

Samples were allowed to coagulate at room temperature for 30 min, then centrifuged at 2000× *g* for 10 min to separate serum, which was stored at −80 °C until ELISA analysis. Storage time did not exceed six months.

Serum sPD-1 and sPD-L1 levels were measured in duplicate using quantitative sandwich ELISA kits, and mean values were used for analysis. Specifically, sPD-1 was quantified using the Human PD-1 sandwich ELISA Kit (#KE00075, Proteintech Group, Inc., Rosemont, IL, USA) with intra-assay and inter-assay coefficients of variation (CV) below 3.7% and 5.8%, respectively. The assay’s limit of detection (LOD) was 43.0 pg/mL per the manufacturer’s specifications. sPD-L1 was measured using the Human PD-L1 sandwich ELISA Kit (#KE00074, Proteintech Group, Inc., Rosemont, IL, USA) with intra-assay and inter-assay CVs below 7.8% and 7.0%, respectively, and an LOD of 0.04 ng/mL. All measurements and readings were performed according to the manufacturer’s instructions.

### 2.4. Statistical Analysis

Statistical analyses were performed using GraphPad Prism software, version 10.4.1 (GraphPad Software, La Jolla, CA, USA). Baseline characteristics and clinical outcomes were summarized as percentages for categorical variables, and as mean ± standard deviation (SD) or median (range) for continuous variables, depending on their distribution. Data normality was assessed using the Shapiro–Wilk test. Median time to recurrence was estimated using the Kaplan–Meier method.

Baseline levels of soluble checkpoint proteins were compared among groups using the non-parametric Kruskal–Wallis test. Longitudinal (pre- vs. post-treatment) changes were evaluated with the Wilcoxon matched-pairs signed-rank test, including only patients with available paired measurements. Percent changes (Δ%) from baseline to post-treatment values were calculated as follows:


$$\Delta \%=\frac{\left(Post-treatment\;value-Baseline\;value\right)}{Baseline\;value}\times 100$$

Univariate logistic regression analyses were performed to assess the associations between baseline levels and Δ (%) of checkpoint inhibitors and clinical endpoints—recurrence for the early-stage cohort and treatment response for the advanced-stage cohort. In addition, in order to apply a biologically relevant threshold of Δ (%), an increase/decrease of >20% was also assessed for association with clinical outcomes, as has been proposed in other analytical studies [15]. Odds ratios (ORs) and corresponding 95% confidence intervals (CIs) were reported.

For the advanced-stage cohort, the relationship between serum sPD-L1 and tumor PD-L1 expression (TPS) was examined using simple linear regression. The Pearson correlation coefficient (*ρ*) and coefficient of determination (*R*^2^) were calculated.

All statistical tests were two-sided, and a *p*-value < 0.05 was considered statistically significant.

## 3. Results

### 3.1. Patients, Baseline Characteristics and Clinical Outcomes

Within a one-year period (December 2019 to December 2020), 25 consecutive patients with early-stage NSCLC who fulfilled all inclusion criteria and none of the exclusion criteria were enrolled in the study. The median age was 67 years (range, 33–84), and the majority were males (n = 15, 60%). All patients underwent lobectomy with lymph node dissection (25, 100%). Most patients had clinical stage I disease (n = 19, 76%) before surgery, while the remaining had stage II (n = 3, 12%) or stage III disease (n = 3, 12%). Postoperative pathological staging revealed 12 patients (48%) with stage I disease, eight (32%) with stage II, and five (20%) with stage III disease. Histological subtypes included 18 cases (72%) of adenocarcinoma and seven (28%) of squamous cell carcinoma. No tissue PD-L1 testing and molecular profiling were performed in the early-stage cohort. Eight patients (32%) received adjuvant platinum-based chemotherapy for four cycles. The recurrence rate was 40% (10 patients), and the median time to recurrence was not reached.

The control group comprised 16 patients enrolled between December 2019 and October 2023. Reasons for admission to the Thoracic Surgery Clinic included rib fractures, thoracic strains, and pneumothorax. The median age was 66 years (range, 45–85), and most were males (n = 9, 56.3%).

The advanced-stage NSCLC cohort included 55 consecutively enrolled patients between December 2019 and November 2023. The median age was 67 years (range, 33–84), with most being males (n = 38, 69.1%). Fifty patients (90.9%) had stage IV disease at diagnosis, while the remaining five (9.1%) had unresectable stage III disease and received first- or second-line systemic therapy. Most patients had adenocarcinoma (n = 35, 63.6%), followed by squamous cell carcinoma (n = 18, 32.7%) and carcinoma not otherwise specified (NOS) (n = 2, 3.6%). The majority had a good ECOG Performance Status (PS) of 0–1 at baseline (n = 46, 83.6%). PD-L1 was variably expressed, with most patients showing high PD-L1 tumor proportion score (TPS ≥ 50%) (n = 32, 58.2%), followed by intermediate expression (TPS 1–49%) (n = 14, 25.5%) and negative expression (TPS ≤ 1%) (n = 9, 16.4%). No patients in the advanced-stage cohort carried targetable first-line mutations (EGFR mutations or ALK rearrangement). Among patients with the adenocarcinoma subtype, two patients carried targetable second-line mutations (KRAS G12C and BRAF V600E, respectively). Most patients received first-line immunotherapy (n = 49, 89.1%), while the remainder received second-line treatment (n = 6, 10.9%). All patients were treated with the anti-PD-1 agent pembrolizumab, administered either as monotherapy or in combination with platinum-based chemotherapy (63.6% and 14%, respectively). The objective response rate (ORR) at six months was 26.9% (14 patients).

The distribution of populations among cohorts was balanced for age (*p* = 0.52), gender (*p* = 0.55), and histological subtype (*p* = 0.82).

Baseline clinicopathological characteristics are summarized in Table 1, and treatment characteristics and clinical outcomes in Table 2.

### 3.2. Soluble PD-L1 and PD-1 Comparison Across Cohorts

**Figure 1:**
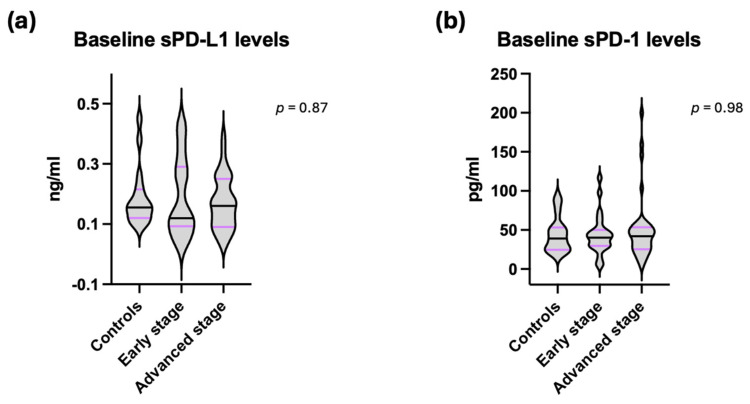
Comparison of (**a**) sPD-L1 and (**b**) sPD-1 levels between healthy controls, early- and advanced-stage non-small cell lung cancer cohorts.

Pre-treatment levels of sPD-L1 and sPD-1 were measured in the control, early-stage, and advanced-stage NSCLC cohorts. Median sPD-L1 levels were 0.15 ng/mL (0.10–0.45) in controls, 0.12 ng/mL (0.04–0.42) in the early-stage cohort, and 0.17 ng/mL (0.03–0.40) in the advanced-stage cohort. Median sPD-1 levels were 39.08 pg/mL (19.17–91.97) in controls, 40.22 pg/mL (4.93–116.68) in the early-stage cohort, and 43.00 pg/mL (4.37–649.78) in the advanced-stage cohort. Differences in median levels among the three groups were not statistically significant for either sPD-L1 or sPD-1 (Figure 1).

Figures are illustrated as violin plots with median (range). The Kruskal–Wallis test for non-normally distributed data was used for comparison of medians.

### 3.3. Longitudinal Fluctuations of sPD-L1 and sPD-1 in the Lung Cancer Cohorts

**Figure 2:**
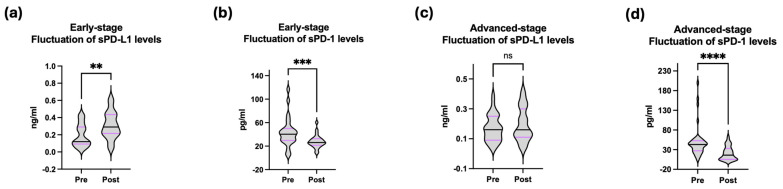
Comparison of pre- and post-treatment sPD-L1 and sPD-1 levels in the early-stage (**a**,**b**) and advanced-stage (**c**,**d**) non-small cell lung cancer cohorts. **: <0.01; ***: <0.001; ****: <0.0001.

To evaluate the longitudinal kinetics of sPD-L1 and sPD-1 during treatment, a pre-treatment versus post-treatment analysis was performed (Figure 2). In the early-stage NSCLC cohort, comparison of baseline and post-surgical soluble checkpoint levels revealed a statistically significant increase in sPD-L1 (*p* = 0.006) and a significant decrease in sPD-1 (*p* < 0.001). In the advanced-stage cohort, sPD-1 levels showed a significant reduction at six months of treatment (*p* < 0.001), while sPD-L1 levels did not differ significantly from post-treatment (*p* = 0.37).

Figures are illustrated as violin plots with median (range). The Wilcoxon ranked-sum test for paired comparisons was used to assess the difference between pre- and post- treatment concentration levels.

### 3.4. Association of sPD-L1 and sPD-1 Levels with Prognosis

The prognostic value of baseline concentrations and early kinetics of soluble PD-L1 and PD-1 was evaluated separately in the early- and advanced-stage NSCLC cohorts (Table 3).

In the early-stage cohort, baseline levels of sPD-L1 and sPD-1 were not significantly associated with recurrence (*p* = 0.87 and *p* = 0.43, respectively). Similarly, the continuous percentage changes (Δ%) of sPD-L1 and sPD-1 from baseline to the post-surgical timepoint were not predictive of recurrence (*p* = 0.29 and *p* = 0.17, respectively). However, when applying a predefined threshold of a >20% increase in sPD-L1, patients exceeding this cutoff had a significantly higher risk of recurrence (OR = 10.29; 95% CI: 1.40–215.20; *p* = 0.019). A >20% decrease in sPD-1 did not demonstrate a significant association with recurrence (*p* = 0.25).

In the advanced-stage cohort, neither baseline sPD-L1 nor baseline sPD-1 levels were significantly associated with response to immunotherapy (*p* = 0.07 and *p* = 0.29, respectively). Likewise, Δ% changes in sPD-L1 (*p* = 0.66) and sPD-1 (*p* = 0.56) were not predictive of treatment response. A >20% decrease in sPD-1 was also not associated with improved response rates (*p* = 0.87).

### 3.5. Correlation of sPD-L1 and Tissue PD-L1 in the Advanced-Stage Lung Cancer Cohort

**Figure 3:**
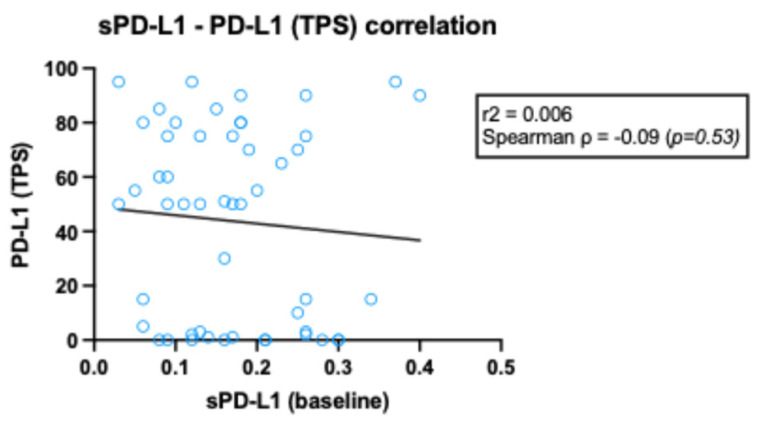
Correlation of baseline sPD-L1 and tissue PD-L1 (per tumor proportional score, TPS) in the advanced-stage non-small cell lung cancer cohort.

A correlation analysis was performed to evaluate the association between tissue PD-L1 expression (TPS) and baseline sPD-L1 levels (Figure 3). No significant correlation was observed between the two biomarkers, with the negative correlation coefficient failing to reach statistical significance (ρ = −0.09, *p* = 0.53; R^2^ = 0.006).

## 4. Discussion

In this prospective study, we evaluated the clinical utility of sPD-L1 and sPD-1 across healthy individuals and patients with early- and advanced-stage NSCLC, focusing on their longitudinal kinetics and associations with clinical outcomes. Our findings reveal several notable observations. First, baseline concentrations of sPD-L1 and sPD-1 did not differ significantly among controls, early-stage, and advanced-stage cohorts. Second, longitudinal analysis identified distinct kinetic patterns in the two NSCLC settings: in early-stage disease, sPD-L1 significantly increased while sPD-1 markedly decreased following surgical resection, whereas in advanced-stage disease, only sPD-1 decreased during immunotherapy, with no significant change observed in sPD-L1 levels. Third, although most baseline and kinetic parameters were not independently predictive of outcomes, a ≥20% postoperative increase in sPD-L1 was associated with a substantially higher risk of recurrence in early-stage patients. Finally, no correlation was found between circulating sPD-L1 concentrations and tumor PD-L1 expression, suggesting biological independence between tissue and soluble checkpoint compartments.

The lack of differences in baseline sPD-L1 levels across controls and NSCLC patients in our cohort contrasts with earlier case-control studies, which reported significantly higher sPD-L1 concentrations in lung cancer patients compared with healthy individuals and even proposed diagnostic cutoffs for NSCLC discrimination [16,17]. This discrepancy may reflect differences in tumor burden, inflammatory milieu, methodological heterogeneity among ELISA platforms, or the relatively small number of controls included in our study. In contrast, non-significant differences in sPD-1 levels between lung cancer patients and healthy populations have also been previously reported [17]. However, in other studies, higher levels of sPD-1 in lung cancer patients, compared with healthy controls, have been reported [18]. Importantly, our data suggest that circulating levels of these soluble molecules, at least at a single time point, may not reflect disease presence or stage with sufficient accuracy to serve as diagnostic markers.

The postoperative increase in sPD-L1 observed in early-stage patients likely reflects subacute immune activation following surgery, including cytokine-driven shedding or alternative splicing of PD-L1 from circulating immune cells. This observation comes in agreement with some reports showing a postoperative flare of the marker, while other studies have demonstrated a significant decline of sPD-L1 post-surgery [18,19]. It has been suggested that stromal, dendritic, and myeloid cells can contribute substantially to circulating PD-L1 pools, particularly in inflammatory contexts. Sharp increases of sPD-L1 may originate from immune or stromal cells via extracellular vesicles, alternative splicing, or proteolytic cleavage, rather than tumor cell shedding alone [12,13,20]. Divergent reports on postoperative sPD-L1 dynamics across studies can be explained by the complex and heterogeneous biology of soluble checkpoint molecules. sPD-L1 originates not only from tumor tissue but also from activated monocytes, dendritic cells, and other stromal or immune populations. Thus, the net postoperative effect reflects the balance between loss of tumor-derived PD-L1 following resection and the concurrent immune activation induced by surgery. Studies sampling very early after surgery commonly report declining levels dominated by tumor removal, whereas samples obtained weeks to months later—during sustained wound-healing-related inflammation or adjuvant chemotherapy—often show increased sPD-L1 driven by cytokine-mediated expression and shedding. Additional variability arises from differences in assay platforms, tumor histology, and perioperative immune responses. Together, these factors explain why postoperative sPD-L1 trajectories vary across cohorts and support the concept that soluble checkpoints reflect systemic immune remodeling rather than tumor burden alone.

The concurrent decline in sPD-1 levels post-surgery is intriguing and may reflect decreased systemic immune exhaustion or reduced circulating T-cell activation following tumor removal. Perioperative dynamics of sPD-1 are also inconclusive in the literature, with some studies showing a decrease of the marker [18], while others describe increases in the postoperative period [21]. Assuming the same limitations in measuring consistently sPD-1 as in the measurement of sPD-L1, the heterogeneous perioperative shifts support the view that soluble PD-1/PD-L1 levels reflect broader systemic immune remodeling in response to surgical intervention, rather than simply mirroring tumor bulk.

In advanced-stage disease, we observed a significant decline in sPD-1 levels during pembrolizumab-based therapy, whereas sPD-L1 levels remained largely stable. The biological interpretation of these dynamics is not straightforward, as published data on soluble checkpoints under ICI treatment are heterogeneous. Recent clinical series have primarily focused on sPD-L1, with lower or decreasing sPD-L1 levels during therapy being associated with durable benefit and improved outcomes in some cohorts [10]. In contrast, Himuro et al. reported that higher or increasing sPD-1 and sPD-L1 concentrations could be seen in patients with clinical benefit, underscoring the complexity of soluble checkpoint biology under immunotherapy [11]. Our finding of declining sPD-1 levels during treatment therefore suggests that soluble PD-1 may capture a different aspect of systemic immune modulation in our cohort. This decrease may reflect dynamic changes in T-cell activation and exhaustion status under PD-1 blockade and highlights the need for further work to clarify how sPD-1 kinetics relate to T-cell activation, exhaustion, and treatment efficacy.

Among the prognostic analyses, the most clinically meaningful observation was that a postoperative increase of more than 20% in sPD-L1 was strongly associated with recurrence in early-stage NSCLC. This threshold-based approach may capture biologically relevant shifts in soluble checkpoint dynamics that are not apparent when examining continuous percent changes. Prior studies evaluating dynamic serum biomarkers, including tumor markers and inflammatory mediators, have similarly shown that predefined relative changes may outperform absolute or continuous measurements in predicting outcomes [15]. To our knowledge, this is among the first studies demonstrating unfavorable outcomes for patients with a significant post-operative increase of sPD-L1, suggesting that establishing clinically meaningful kinetic thresholds may add to the prognostic value of the marker. Although our finding requires external validation, it highlights a potential role for postoperative soluble PD-L1 monitoring as an early alert for recurrence risk and may help identify patients who could benefit from intensified surveillance or adjuvant therapy, especially in current approved perioperative immunotherapy regimens.

The lack of prognostic value for baseline or dynamic sPD-L1 in our advanced-stage cohort is consistent with previous reports showing no association between sPD-L1 levels and early treatment response [17]. However, other studies have demonstrated prognostic relevance, with lower baseline sPD-L1 linked to durable benefit [10], and higher or rising sPD-L1 during treatment associated with clinical benefit in selected cohorts [11]. In a recent meta-analysis by Shimizu et al., including advanced NSCLC sPD-L1 kinetic studies, an increase of sPD-L1 was not significantly associated with clinical outcomes (OS (HR = 1.25; CI: 0.52–3.02)/PFS (HR = 1.42; CI: 0.61–3.30)) [22].

A subset of the advanced-stage cohort included in the present analysis has been previously reported in a survival-focused study evaluating the prognostic significance of soluble immune checkpoint molecules in ICI-treated NSCLC [23]. That work primarily assessed baseline and progression-timepoint biomarker levels using ROC-derived thresholds in relation to overall survival outcomes. In contrast, the current study was designed to investigate longitudinal kinetics across disease stages, employing fixed on-treatment landmarks and dynamic percentage changes, and to explore associations with early treatment response rather than cumulative survival. Notably, while baseline sPD-L1 was previously shown by our group to have prognostic relevance for survival in advanced-stage disease, the present kinetic-focused analysis did not demonstrate significant associations between baseline or dynamic sPD-L1 levels and early treatment response. This divergence likely reflects differences in clinical endpoints, sampling strategies, and statistical modeling approaches, suggesting that sPD-L1 may retain prognostic value for long-term outcomes while exhibiting limited predictive capacity for early radiologic response. Together, these complementary analyses highlight the context-dependent biological behavior of soluble immune checkpoint biomarkers.

The absence of correlation between tissue PD-L1 (TPS) and circulating sPD-L1 further reinforces the notion that these compartments capture distinct biological processes and is in concordance with previous findings [24]. Tumor PD-L1 expression reflects the spatially restricted and heterogeneously distributed presence of membrane-bound ligand within tumor cells or immune infiltrates, whereas circulating sPD-L1 likely arises from a broader spectrum of cellular sources, including peripheral immune cells and stromal components [12,13]. Additionally, exosomal PD-L1—biologically active and highly reflective of tumor–immune interactions—may not be reliably captured by standard ELISA assays, further contributing to the divergence between tissue and serum measurements. As such, sPD-L1 should be viewed not as a direct surrogate of tissue PD-L1 but rather as an independent immunological biomarker with its own potential prognostic role.

This study has several limitations. First, the sample size—particularly in the early-stage cohort—is modest, limiting statistical power and precluding multivariable analyses. Second, sPD-1 and sPD-L1 were measured at only two timepoints; more frequent sampling might better capture dynamic patterns and improve predictive accuracy. Third, the advanced-stage cohort was assessed only at six months, whereas earlier on-treatment timepoints (e.g., post-cycle 1 or 2) might be more informative for predicting immunotherapy benefit. Fourth, ELISA-based measurements of soluble immune checkpoints lack assay harmonization across studies, and absolute values may vary substantially depending on assay design. Finally, this was a single-center study, and findings may not be generalizable across populations with different biological or treatment characteristics.

Future research should focus on integrating soluble checkpoint dynamics with complementary biomarkers, such as circulating tumor DNA, cytokine signatures, exosomal PD-L1, T-cell activation markers, and pharmacokinetic parameters of immune checkpoint inhibitors. Validation in larger, multi-center cohorts and evaluation at earlier treatment timepoints may further clarify the predictive value of these biomarkers. Ultimately, a multi-analyte approach may provide more robust prognostic and predictive information than any single biomarker alone.

## 5. Conclusions

In conclusion, our study provides a comprehensive evaluation of sPD-L1 and sPD-1 dynamics across different NSCLC settings. While baseline levels do not discriminate between disease states, postoperative increases in sPD-L1 may predict recurrence in early-stage NSCLC, and declining sPD-1 levels during immunotherapy may reflect treatment-related immune modulation. These findings highlight the potential clinical applications of soluble immune checkpoint monitoring, while also underscoring the need for further investigation to define their role in personalized immuno-oncology.

## Figures and Tables

**Table 1:** Baseline clinicopathological characteristics of the healthy controls, early- and advanced-stage non-small cell lung cancer cohorts.

Clinicopathological Parameters	Control Group (n = 16), %	Early Stage (n = 25), %	Advanced Stage (n = 55), %	*p*-Value (Statistical Test)
Age *	66 (45–85)	67 (54–81)	67 (33–48)	0.52 (Kruskal-Wallis)
Males	9 (56.3)	15 (60)	38 (69.1)	0.55 (Chi-square)
cStage				
I	NA	19 (76)	-	-
II		3 (12)	-	
III		3 (12)	5 (9.1)	
IV		-	50 (90.9)	
pStage				
I	NA	12 (48)	NA	-
II		8 (32)		
III		5 (20)		
Histological subtype				
Adenocarcinoma	NA	18 (72)	35 (63.6)	0.82 (Fisher’s exact test)
Squamous		7 (28)	18 (32.7)	
NOS		-	2 (3.6)	
ECOG PS				
0–1	NA	NA	46 (83.6)	-
≥2			9 (16.4)	
PD-L1 (TPS)				
≤1%	NA	NA	9 (16.4)	-
1–49%			14 (25.5)	
≥50%			32 (58.2)	

cStage: Clinical stage; ECOG PS: Eastern Cooperative Oncology Group Performance Status; NA: not applicable; NOS: not otherwise specified; pStage: Pathological stage; PD-L1: programmed-death ligand-1; TPS: tumor proportion score; * median (range).

**Table 2:** Treatment and outcomes of early and advanced-stage non-small cell lung cancer cohorts.

NSCLC Cohorts	Treatment and Outcomes	N (%)
Early stage (n = 25)	Adjuvant chemotherapy	8 (32)
	Recurrence rate	10 (40)
	Median time to recurrence (months)	NR
Advanced stage (n = 55)	Line of treatment	1 L: 49 (89.1) 2 L: 6 (10.9)
	Pembrolizumab and platinum-based chemotherapy	35 (63.6)
	Pembrolizumab monotherapy	20 (36.4)
	ORR (6 months) ^†^	14 (26.9)

L: line of treatment; N: number; NR: not reached; NSCLC: non-small cell lung cancer; ORR: objective response rate; ^†^ number of patients with available data: 52.

**Table 3:** Univariate analysis for the prognostic value of baseline levels and kinetics of soluble PD-1 and PD-L1 in the early- and advanced-stage non-small cell lung cancer cohorts.

Parameter	NSCLC Setting	Endpoint	OR	95% CI	*p*-Value
Baseline sPD-L1	Early stage	Recurrence	0.586	0.00–327.50	0.87
	Advanced stage	Response	0.001	1.44–1.97	0.07
Δ (%) sPD-L1	Early stage	Recurrence	1.002	0.99–1.01	0.29
	Advanced stage	Response	1.001	0.99–1.00	0.66
Δ (%) sPD-L1 > 20% increase	Early stage	Recurrence	10.29	1.40–215.20	<0.05
Baseline sPD-1	Early stage	Recurrence	1.013	0.98–1.05	0.43
	Advanced stage	Response	1.005	0.99–1.02	0.29
Δ (%) sPD-1	Early stage	Recurrence	0.988	0.96–1.00	0.17
	Advanced stage	Response	0.996	0.98–1.01	0.56
Δ (%) sPD-1 > 20% decrease	Early stage	Recurrence	2.667	0.52–16.37	0.25
	Advanced stage	Response	1.167	0.21–9.27	0.87

CI: confidence interval; NSCLC: non-small cell lung cancer; OR: odds ratio; sPD-1: Soluble PD-1; sPD-L1: Soluble PD-L1; Δ (%): percent change.

## Data Availability

Raw data are included in the Supplementary Materials. Statistical analysis (.prism documents) is available upon request to the corresponding author (via email to itrontzas@med.uoa.gr).

## Supplementary Materials

The following supporting information can be downloaded at: https://www.mdpi.com/article/10.3390/jpm16040203/s1, File S1: Cohorts raw data HIPAA de-identified.
